# The Effect of Chemical Degradation and Polishing on the Gloss of Composite Dental Materials

**DOI:** 10.3390/ma16103727

**Published:** 2023-05-14

**Authors:** Ružica Zovko, Stipo Cvitanović, Mirela Mabić, Zdenko Šarac, Anka Ćorić, Domagoj Glavina, Kristina Goršeta

**Affiliations:** 1 Study of Dental Medicine, School of Medicine, University of Mostar, 80000 Mostar, Bosnia and Herzegovina; stipo.cvitanovic@gmail.com (S.C.); zdenko.sarac@mef.sum.ba (Z.Š.); coricanka1@gmail.com (A.Ć.); 2 Health Care Center Mostar, 80000 Mostar, Bosnia and Herzegovina; 3 Health Care Center Prozor-Rama, 88440 Prozor-Rama, Bosnia and Herzegovina; 4 Faculty of Economics, University of Mostar, 88000 Mostar, Bosnia and Herzegovina; mirela.mabic@gmail.com; 5 School of Dental Medicine, University of Zagreb, 10000 Zagreb, Croatia; glavina@sfzg.hr

**Keywords:** composites, chemical degradation, polishing, gloss

## Abstract

This aim of this study was to investigate surface gloss changes of different composite dental materials after chemical degradation or polishing. Five different composites were used (Evetric, GrandioSO, Admira Fusion, Filtek Z550, Dynamic Plus). The gloss of the tested material was measured with a glossmeter before and after chemical degradation in different acidic beverages. Statistical analysis was performed using a t-test for dependent samples, ANOVA, and a post hoc test. For comparison between groups, a level of significance was set at 0.05. Initial gloss values ranged from 51 to 93 at baseline to 32 to 81 after chemical degradation. The highest values were obtained for Dynamic Plus (93.5 GU) and GrandioSO (77.8 GU), followed by Admira Fusion (82 GU) and Filtek Z550 (70.5 GU). Evetric showed the lowest initial gloss values. After acidic exposures, the gloss measurements revealed different patterns of surface degradation. The results showed that the gloss of the samples decreased with time regardless of the treatment. The interaction between chemical-erosive beverages and the composite could lead to a decrease in the surface gloss of the composite restoration. The nanohybrid composite showed less gloss changes under acidic conditions, suggesting that it is more suitable for anterior restorations.

## 1. Introduction

Aesthetic restorations are indeed one of the greatest challenges in clinical practice today. Many improvements in the aesthetic properties of composites, such as color, translucency, and opalescence, have resulted in better aesthetic properties and a more natural appearance [1]. Surface gloss plays an important role in the aesthetic appearance of composites. The high gloss of the composite also reduces the effect of a color difference between the resin restoration and the surrounding hard dental tissue [2].

The surface quality of the restoration is one of the most important factors in fulfilling aesthetics and achieving better clinical success. The ability of the material to reflect direct light is directly related to the surface quality. This optical phenomenon is referred to as gloss or reflective capacity. Surface texture of dental materials is often measured by gloss [3]. Modern composites can achieve a better gloss effect if an adequate polishing sequence is applied [4]. The gloss directly depends on the roughness of the surface and is the result of the geometric distribution of the light that is reflected on the surface [5]. After polishing, the highest shine is obtained, and it is not stable but changes over time [6]. Research conducted so far on the impact of polishing on the quality of the surface shows a decrease in gloss and an increase in the roughness of various composites, and this depends largely on the type of material [6,7,8,9,10]. For composites that are in the anterior region of the mouth, it is important to mimic the dental enamel and to maintain that gloss over time despite exposure to different conditions in the mouth. Differences between the gloss of the restoration and the surrounding enamel are clinically important because the human eye can easily detect differences in gloss even when the colors are matched. On the other hand, high gloss reduces the color change effect, because the color of the reflected light is more dominant than the color of the underlying composite material (4). High surface gloss is correlated with the aesthetic appearance of the composite and improving the aesthetics of the resin composite restorations [11]. The surface gloss of the composite material depends on the filler content, shape, size, and interparticle spacing, as well as the monomer type and filler-matrix bonding [12]. Composites with small, spherical particles tended to show smoother/glossier surfaces. Suprananofilled/nanofilled/microfilled composites tended to show the smoothest/glossiest surfaces due to smaller particle size [13]. Nanofilled and nanohybrid composites were launched to achieve a better polishing possibility combined with better gloss retention compared to classic microhybrid resin material. Various factors affect the change in surface quality during normal oral function. The oral conditions limit the longevity of restorative materials due to the consumption of acidic drinks, degradation caused by oral fluid components, and mechanical wear. Finishing and polishing also affect surface quality. The surface of composite filling plays a most important role in the degradation of material through absorption and adsorption of oral fluid components, which could lead to softening of the material matrix [13]. Therefore, degradation will firstly become obvious from altered surface properties of a resin composite, followed later by changes in bulk properties [12,13]. Acidic beverages are very popular among adolescents. Such soft drinks are acidic and can produce erosion of teeth as well as resin composites and influence aesthetic properties of material [14].

This study aimed to investigate the gloss properties of various type of composites after exposure to acidic soft drinks and to investigate the effect of different polishing techniques on the surface gloss.

## 2. Materials and Methods

### 2.1. Materials

Five types of composites were investigated in this study: (1) Evetric (Ivoclar Vivadent), (2) GrandioSO (VOCO, Cuxahaven, Germany), (3) Admira Fusion (VOCO, Cuxahaven, Germany), (4) Filtek Z550 (3M ESPE, Seefeld, Germany), (5) Dynamic Plus (President Dental, München, Germany).

### 2.2. Instruments

Surface gloss (GU) was measured with a gloss meter (Gloss checker IG-331, HORIBA Ltd., Minami-ku, Kyoto, Japan). Baseline gloss value and final gloss value of the composites were recorded through gloss meter. The measurements were taken in the middle of the specimens. Three measurements were made per each sample at light incidence and reflection angles of 60° relative to the vertical axis, with the sample rotated 90° each time. The measuring field was 2 mm × 1 mm. The six readings were averaged to obtain a single value for each sample. The specimen was covered with a black shield to exclude ambient interferences [6,15]. Values were expressed in gloss units (GU). Acidity of liquids was measured by pH meter (HI 8014, Hanna Instruments, Bioblock Scientific, Illkirch, France). Standard buffer solution (Solutions tampons techniques 2 X pH = 4; 2 X pH = 7; 2 X pH = 10 (25 °C), 10 X 6 ampoules: L 4998; Cat. No. 93150, Biobloch Scientific) with nominal pH values from 4.0 to 7.0 was used to calibrate the pH meter with an accuracy of 0.01 units.

### 2.3. Specimen Preparation—Protocol

Composite material samples were prepared in accordance with the manufacturer’s instructions. Each group consisted of six specimens. Unpolymerized composite materials (all A2 shades) were placed into Teflon mold (width 5 mm, thickness 2 mm). Mylar strips (Henry Schein, Melville, NY, USA) were placed over unpolymerized composite surface and lightly pressed with a slide glass using finger pressure for 30 s in order to standardize the surface of the samples, extrude the excess of material, and prevent formation of an oxygen inhibition layer. The samples were polymerized for 40 s with a polymerization unit (Bluephase G2, Ivoclar Vivadent, Liechtenstein) in direct contact with the slide glass. The output energy of the curing light was 1200 mW/cm^2^. Five minutes after hardening, the samples were taken out from the mold. The edge flashing was gently removed. The samples were immersed in three different solutions: Coca-Cola, Ice Tee, and distilled water (as the control group). All samples were stored in an incubator at a temperature of 37 ˚C for 30 days according to Kusuma Yulianto HD [13]. The other three groups (n = 6) underwent different surface treatments: two of them were polished and one served as the control group without any treatment. The surface of the specimens was progressively polished under water cooling with polishing discs (500 and 1200 grit; FEPA, Struers, Ballerup, Denmark) on an automatic polishing machine (RotoPol 11, Struers, Ballerup, Denmark). This procedure was performed in order to achieve a parallel surface for measuring the surface gloss. On each sample, the gloss was measured six times: twice at three different locations of the sample.

### 2.4. Statistical Analysis

Results are expressed as mean and standard deviation. The t-test for dependent samples, ANOVA, and post hoc test were used for testing the significance of observed differences. The significance of the obtained results was analyzed at the significance levels of 0.05 and 0.001.

## 3. Results

**Figure 1:**
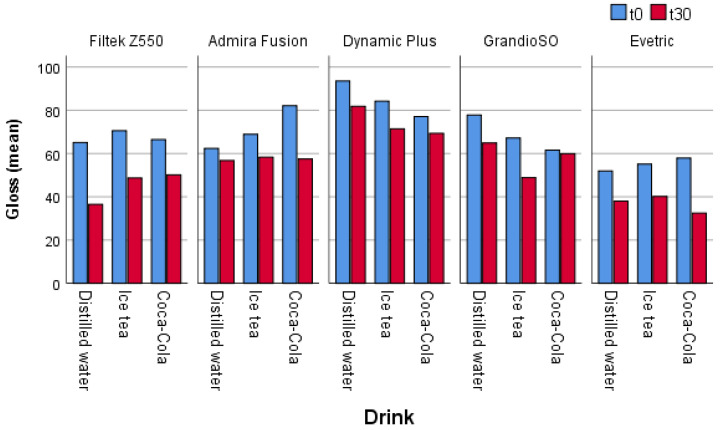
The effect of different pH values of drinks on the gloss of restorative dental materials after 30 days.

A comparison of the gloss of the different restorative materials exposed to beverages of different pH values in all cases (in all samples) shows a reduction in gloss (Figure 1), and in most cases it is a statistically significant reduction in gloss (Table 1).

In the samples treated with distilled water, a statistically significant difference in gloss level after 30 days was found in four out of the five materials analyzed. The greatest reduction in gloss in the distilled water samples was found in the material Filtek Z550, while the smallest reduction was found in the material Admira Fusion (Table 1).

In the samples treated with iced tea, a statistically significant difference in the level of gloss after 30 days was found in all materials. The greatest reduction in gloss in iced tea samples was found in the material Filtek Z550, while the smallest reduction was found in the material Admira Fusion (Table 1).

In the samples treated with Coca-Cola, a statistically significant difference in gloss level after 30 days was found in three of the five analyzed materials. The greatest reduction in gloss in Coca-Cola samples was found in Evetric, while the smallest reduction was found in GrandioSO (Table 1).

**Figure 2:**
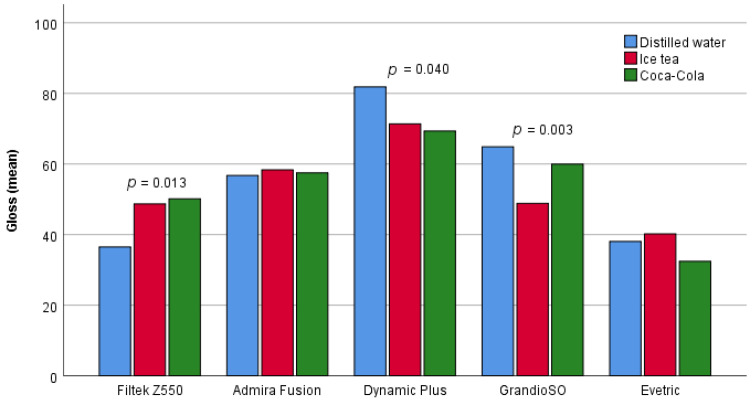
Comparison of the gloss of samples treated with different pH values drinks after 30 days.

Statistically significant differences in the shine of the material after 30 days with regard to the drink containing the samples were found in three materials (Figure 2). For Filtek Z550 materials, statistically significant differences in gloss were found between samples that were in distilled water and samples that were in iced tea (*p* = 0.039), or samples that were in Coca-Cola (*p* = 0.019). Samples of Filtek Z550 material that were in distilled water after 30 days had a significantly lower gloss than samples that were in iced tea and Coca-Cola.

For Dynamic Plus materials, a statistically significant difference in gloss was found between the samples that were in distilled water and the samples that were in Coca-Cola (*p* = 0.046). The samples of Dynamic Plus material that were in distilled water, after 30 days, had a significantly higher glow than samples that were in Coca-Cola.

The samples of GrandioSO material that were in iced tea, after 30 days, had a significantly lower shine than the samples that were in distilled water and Coca-Cola.

**Figure 3:**
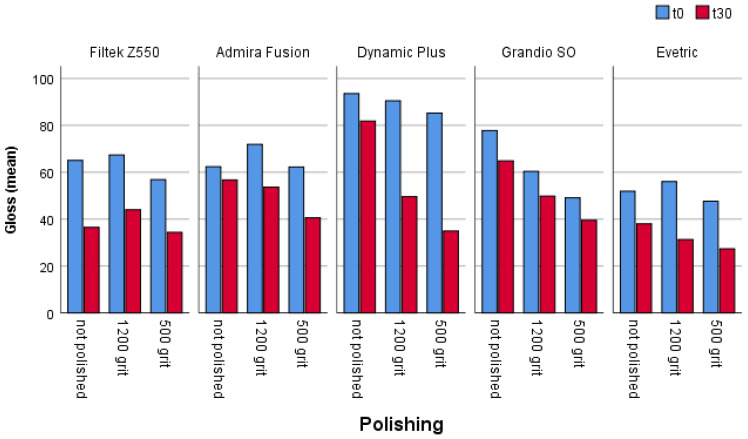
The effect of polishing on the gloss of restorative dental materials.

A comparison of the gloss of the different restorative materials that are not polished (control groups) and those that are polished with different discs in all cases (in all samples) shows a reduction in gloss (Figure 3), and in most cases it is a statistically significant reduction (Table 2).

In the non-polished samples, a statistically significant difference in the gloss level after 30 days was found in four of the five materials analyzed. The greatest reduction in gloss in these samples was found in the Filtek Z550 material, while the least reduction was found in the Admira Fusion material (Table 2).

A statistically significant difference in the gloss level after 30 days was found in the samples polished with a 1200 grit disk for all analyzed materials. The greatest reduction in gloss in these samples was found in the Dynamic Plus material, while the least reduction was found in the GrandioSO material (Table 2).

In 500 grit polished disc samples, a statistically significant difference in the gloss level after 30 days was found in all the analyzed materials. The greatest reduction in gloss was found in Dynamic Plus (58%), while the smallest reduction was found in GrandioSO and Evetric (Table 2).

**Figure 4:**
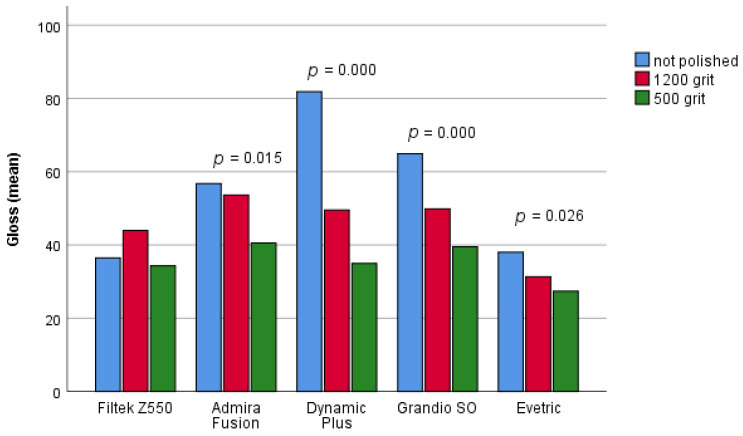
Comparison of the gloss of unpolished and polished samples after 30 days.

Statistically significant differences in the material gloss after 30 days with regard to polishing were found in four composites (Figure 4). Post-hoc analysis showed a significant difference between the unpolished samples and those polished with a 500-grit disc, in all four composites [p (Admira Fusion) = 0.016; p (Dynamic Plus) = 0.000; p (GrandioSO) = 0.000; p (Evetric) = 0.021]. In all four materials, the unpolished samples had a significantly higher shine after 30 days compared to the samples polished with 500 grit discs. Furthermore, a significant difference between unpolished samples and samples polished with a 1200-grit disc was found in the samples of Dynamic Plus material (*p* = 0.000) and GrandioSO (*p* = 0.002). The non-polished samples of these two materials had a significantly higher gloss after 30 days compared to the samples polished with 1200 grit discs. Statistically significant differences in the gloss of polished samples with regard to the type of discs were found in samples of GrandioSO material (*p* = 0.038). The samples polished with a 1200 grit disk had a significantly greater gloss after 30 days than the samples polished with a 500-grit disk.

## 4. Discussion

Due to high aesthetic demand, resin composite material is the most common material of choice in restorative dentistry. Previous studies have been reported that surface topography of filling could be affected with different solutions in the oral cavity. Erosion could be caused by some gastrointestinal disorders or as side effects of some drugs. Moreover, erosion could occur due to some acidic food or drinks. Most of the soft drinks have phosphoric acid as an ingredient [16]. Restorative composite materials are subjected to a variety of factors including dietary habits, food, and liquid coloring agents, chemical, thermal, and mechanical processes, oral hygiene, which all may affect the properties of the materials over time [17]. For achieving good aesthetic and clinical success, it is mandatory to obtain proper surface gloss and smoothness. Rough surfaces will promote plaque retention and plaque formation. It is knownthat the high-energy surface collects more debris, allows more bacteria to adhere, and promotes plaque formation. Smooth surfaces play an importantrole in the aesthetic appearance of the composite. The initial surface gloss of the nanofilled composite is improved, mainly due to the polishable particle size and a higher percentage by volume of nanofillers in the resin matrix. As a polymer-based material, resin-based composites may undergo degradation when exposed to the oral environment. The process of degradation may lead to a reduction in surface topography properties [14].

Through this research, it was found that the liquid in which the composite material was carried out had an impact on its gloss. Erosion describes the process of gradually destroying the surface of something, usually by chemical or electrolytic process [17]. The study was conducted in three liquids: distilled water and acidic drinks such as Coca-Cola and iced tea.

Different studies confirm obtained results [18]. Francisconi et al. showed that under the influence of the low pH of beverages on the restorative material, the material surface and physical and chemical properties change [18]. Through this investigation of the influence of given liquids on the composite material, it was found that the gloss decreases with time in all tested materials groups, and it depended on the liquid it was in. The greatest reduction in gloss was confirmed for the composites residing in Coca-Cola.

Such influence of Coca-Cola is thought to be due to the presence of acids, and the highest phosphoric acid contained in the composition of this drink affects the low pH = 2.5 [19]. There is also the influence of phosphoric acid on the organic matrix, which softens and thus leads to a change in light refraction and consequently a decrease in gloss [20,21].

Although surface polishing has been shown to be strongly related to surface gloss, this study shows that other aging protocols, such as acid erosion from beverage immersion, may have a greater impact on surface gloss.

Previously, it has been confirmed that when the composites are exposed to various environmental influences, there is a certain reduction in the gloss of the material [22]. It could be that the difference occurs due to the difference in size and shape of the filler. Lee et al. proved that the influence of the given environment had no effect on some composite materials, and that all others showed a change in gloss and surface [23]. In this study, the surface gloss values of Dynamic Plus remained above 80 GU with less than a 10% reduction after acidic degradation. On the other side, the baseline surface gloss values of Evetric started above 57 GU but drastically dropped by more than 50% after degradation in Coke.

In six of the ten studies, microfilled composites were compared to nanofilled and/or suprananofilled composites, with three showing that the nanofilled composites have glossier surfaces, and in one study the nanofilled surface was also smoother than the microfilled surface. Composites with small, spherical particles tend to have smoother, glossier surfaces and are more wear resistant than resin composites with larger or irregular particles. Several studies showed higher roughness and lower gloss after abrasion with a toothbrush [14,23,24,25,26,27,28].

Compared with the polishing factors on the gloss of the material over a period of time, the results were found similar to the previous studies. It has been confirmed that more polished the material is, the smaller is the material gloss. Some studies showed that the effectiveness of the polishing system depended on the material rather than the polishing system [14]. Composites with small, spherical particles tend to have smoother, glossier surfaces and are more wear resistant than resin composites with larger or irregular particles [14,18,22].

In this study, the composite Filtek Z550 also showed the highest gloss when polished with a 1200 grit discs. In contrast, the Evetric, Admira Fusion, and Dynamic Plus composites showed less gloss loss when not polished compared to the polished samples, but the difference was not statistically significant.

Previous studies reported that suprananofilled and nanofilled materials have high gloss in the range from 52 to 92 GU. Moreover, composites with spherical fillers showed the highest gloss value [12]. Camassari et al. concluded that biodegradation increased the surface roughness and decreased the GU [29]. Salerno and coworkers showed that different conditions of air polishing could increase surface roughness of dental restoration [30].

Gurgan et al. found that G-ænial A’chord has excellent optical properties, which is supported by the improved polishing properties of these composite resins [31]. Even composites that belong to the same category can have a wide range of filler sizes and shapes, different volume or weight percentages of fillers, or different types of resin matrices, making it very difficult to compare one composite to another to see the effects of a particular component in resin composites on optical properties such as surface gloss [14]. Ormocers are composed of ceramic polysiloxane and combine organic and inorganic components at the nanoscopic level [14]. Batista et al. showed that nanohybrid composites exhibited lower surface roughness and higher gloss values than the other composites tested [32]. Admira Fusion as an ormocer-based nanohybrid composite showed quite stable behavior in this study. 

Lassila et al. showed that samples polished with 4000-grit paper exhibited higher gloss (93 GU) than samples polished with 320-grit paper [33]. Similar results were also obtained in this study.

## 5. Conclusions

The interaction between chemically erosive beverages and the composite could lead to a decrease in surface gloss and surface quality, which in turn affects the aesthetic properties of the composite restoration. The type of composite could have significant effects on the gloss of resin composites. The nanohybrid composite shows the best behavior (less gloss changes) under acidic conditions, suggesting that it is more suitable for use in anterior restorations. It could be concluded that the type of the composite material is crucial for its long-term surface properties and that diet and consumption of acidic drinks will influence the material surface properties.

For further research, it would be interesting to correlate the gloss values with the surface roughness values and possibly extrapolate some clinically important points.

## Figures and Tables

**Table 1:** The mean gloss (GU) of restorative dental materials after 30 days treated with the different pH values of drinks.

		T0 (GU)		T30 (GU)				
		M	SD	M	SD	MD	%	*p*
DW	Filtek Z550	65.08	14.07	36.48	10.55	28.59	43.94	0.005 **
	Admira Fusion	62.38	15.82	56.75	15.52	5.63	9.02	0.542
	Dynamic Plus	93.51	4.43	81.82	7.03	11.68	12.49	0.001 **
	GrandioSO	77.79	10.61	64.88	10.67	12.91	16.59	0.007 *
	Evetric	51.91	6.72	38.03	9.28	13.87	26.72	0.012 *
IT	Filtek Z550	70.53	8.69	48.69	11.08	21.83	30.95	0.001 **
	Admira Fusion	68.88	11.53	58.34	6.43	10.54	15.30	0.048 *
	Dynamic Plus	84.18	10.03	71.38	8.60	12.80	15.20	0.009 *
	GrandioSO	67.15	11.44	48.83	9.92	18.32	27.28	0.005 *
	Evetric	55.13	8.13	40.19	6.97	14.94	27.10	0.010 *
CC	Filtek Z550	66.45	11.24	50.15	7.71	12.30	18.51	0.001 **
	Admira Fusion	82.10	7.57	57.48	12.23	24.61	29.97	0.001 **
	Dynamic Plus	77.11	9.71	69.34	14.29	7.77	10.07	0.099
	GrandioSO	61.59	6.08	59.93	5.19	1.66	2.69	0.510
	Evetric	57.92	9.14	32.44	7.96	25.48	43.99	0.000 **

DW—Distilled water; IT—Iced tea; CC—Coca-Cola; T0—first day; T30—30th day; M—mean; SD—standard deviation; MD—mean difference; GU—gloss unit; * *p* ≤ 0.05; ** *p* ≤ 0.001.

**Table 2:** The mean gloss of not polished and (with different discs) polished restorative dental materials after 30 days.

		T0 (GU)		T30 (GU)				
		M	SD	M	SD	MD	%	*p*
np	Filtek Z550	65.08	14.07	36.48	10.55	28.59	43.93	0.005 *
	Admira Fusion	62.38	15.82	56.75	15.52	5.63	9.02	0.542
	Dynamic Plus	93.51	4.43	81.82	7.03	11.68	12.49	0.001 **
	GrandioSO	77.79	10.61	64.88	10.67	12.91	16.59	0.007 *
	Evetric	51.91	6.72	38.03	9.28	13.88	26.74	0.012 *
1200 gd	Filtek Z550	67.36	12.31	43.99	14.88	23.37	34.69	0.014 *
	Admira Fusion	71.85	6.82	53.63	9.82	18.22	25.35	0.004 *
	Dynamic Plus	90.47	10.72	49.56	13.67	40.92	45.23	0.000 **
	GrandioSO	60.35	8.84	49.83	5.32	10.52	17.43	0.039 *
	Evetric	56.03	10.34	31.30	7.95	24.73	44.14	0.001 **
500 gd	Filtek Z550	56.86	10.18	34.34	10.99	22.51	39.59	0.001 **
	Admira Fusion	62.26	5.78	40.54	7.53	21.72	34.88	0.000 **
	Dynamic Plus	85.19	8.71	34.95	19.78	50.24	58.97	0.000 **
	GrandioSO	49.08	9.08	39.52	8.07	9.56	19.47	0.025 *
	Evetric	47.64	9.79	27.37	5.91	20.27	2.10	0.001 **

np—not polished; 1200 gd—polished with a 1200 grit disc; 500 gd—polished with a 500—grit disc; T0—first day; T30—30th day; M—mean; SD—standard deviation; MD—mean difference; GU—gloss unit; * *p* ≤ 0.05; ** *p* ≤ 0.001.

## Data Availability

The datasets generated during and/or analyzed during the current study are available from the corresponding author on reasonable request.
